# A wearable and sensitive graphene-cotton based pressure sensor for human physiological signals monitoring

**DOI:** 10.1038/s41598-019-50997-1

**Published:** 2019-10-08

**Authors:** Ping Li, Libo Zhao, Zhuangde Jiang, Mingzhi Yu, Zhen Li, Xiangyang Zhou, Yulong Zhao

**Affiliations:** 1 State Key Laboratory for Manufacturing Systems Engineering, International Joint Laboratory for Micro/Nano Manufacturing and Measurement Technologies, Collaborative Innovation Center of Suzhou Nano Science and Technology, Xi’an Jiaotong University, Xi’an, 710049 China; 2 School of Mechanical Engineering, Xi’an Jiaotong University, Xi’an, 710049 China; 3 School of Instrumentation Science and Opto-electronics Engineering, Beihang University, Beijing, 100191 China

**Keywords:** Physical examination, Mechanical and structural properties and devices

## Abstract

Cotton fiber is the most commonly used fabric in textiles and clothing. As compared to inorganic materials like foam, sponge and paper, cotton fibers boast higher levels of flexibility and toughness, which makes it more durable and be better integrated with clothes. In this study, a conductive cotton fiber material modified by reduced graphene oxide (rGO) was prepared, and applied in pressure sensor. The highest sensitivity of the pressure sensor constructed is 0.21 kPa^−1^, and the pressure range covers up to 500 kPa, which demonstrates a combination of fine sensitivity and broader pressure range. The pressure sensor developed in this study demonstrates great performance in real-time monitoring of human physiological signals like pulse, breath rate and speech recognition, boasting great application value in wearable electronics and smart clothing.

## Introduction

With the rapid development of robotics technology and wearable electronics in recent years, flexible pressure sensors widely applied in e-skins and wearable devices have come under the spotlight of a great number of researchers^1–11^. A variety of pressure sensors have appeared with different flexible materials. In these sensors, organic polymers like PDMS^12–14^, rubber^2^, P(VDF-TrFE)^15,16^ are commonly used materials owing to their natural flexibility and transparency. Meanwhile, inorganic materials like foam^17–19^, sponge^20,21^, paper^6^ and textiles^22,23^ serve as active materials for flexible pressure sensors due to their high-efficiency, low cost, and the fact that they can be easily manufactured. As is commonly known, most of our clothes are made of cotton fibers. Compared with inorganic materials mentioned above, cotton fiber based material boasts higher levels of flexibility and toughness, which makes it durable and the most commonly wearable material. In previous studies, functionalized cotton textiles have been employed as a wearable platform in many applications^24–27^.

Recently, owing to its advantageous features, the unique three-dimensional (3D) microstructure based devices have been widely studied^28,29^. Lou *et al*. reported an rGO wrapped P(VDF-TrFE) 3D nanofibers based pressure sensor, whose measuring sensitivity was 15.6 kPa^−1^ and the detection limit was as low as 1.2 Pa^30^. Pan *et al*. fabricated an ultra-sensitive pressure sensor based on 3D hollow-sphere microstructure polypyrrole film, whose measuring sensitivity reached 133.1 kPa^−1^ and detection limit was below 1 Pa^28^. The 3D porous or networks structure forms a 3D conducting path to produce sensitive response to external pressure. The cotton fibers can form a 3D-network microstructure, and cotton is the most commonly used material in textile production, which facilitates its integration with clothes and wearable devices. In this study, by virtue of a simple dipping and annealing process, a conductive cotton fibers material modified by rGO nanosheets was prepared and then used to fabricate a novel flexible pressure sensor. The pressure sensor proposed in this study can be easily fabricated and highly sensitive (0.21 kPa^−1^ in 0–2 kPa and 0.0368 kPa^−1^ in 2–20 kPa) with a broad pressure range spanning up to 500 kPa (see Supporting Information). The response to loading-unloading cycles of external pressures show good reproducibility and stability. The detection of human physiologic signals (such as wrist pulse and respiration rate) was achieved by virtue of high signal-to-noise ratio (SNR). These results show that the pressure sensor based on rGO modified cotton proposed in this study boasts great potential in application in wearable electronics especially in smart clothing.

## Ressults and Discussion

**Figure 1:**
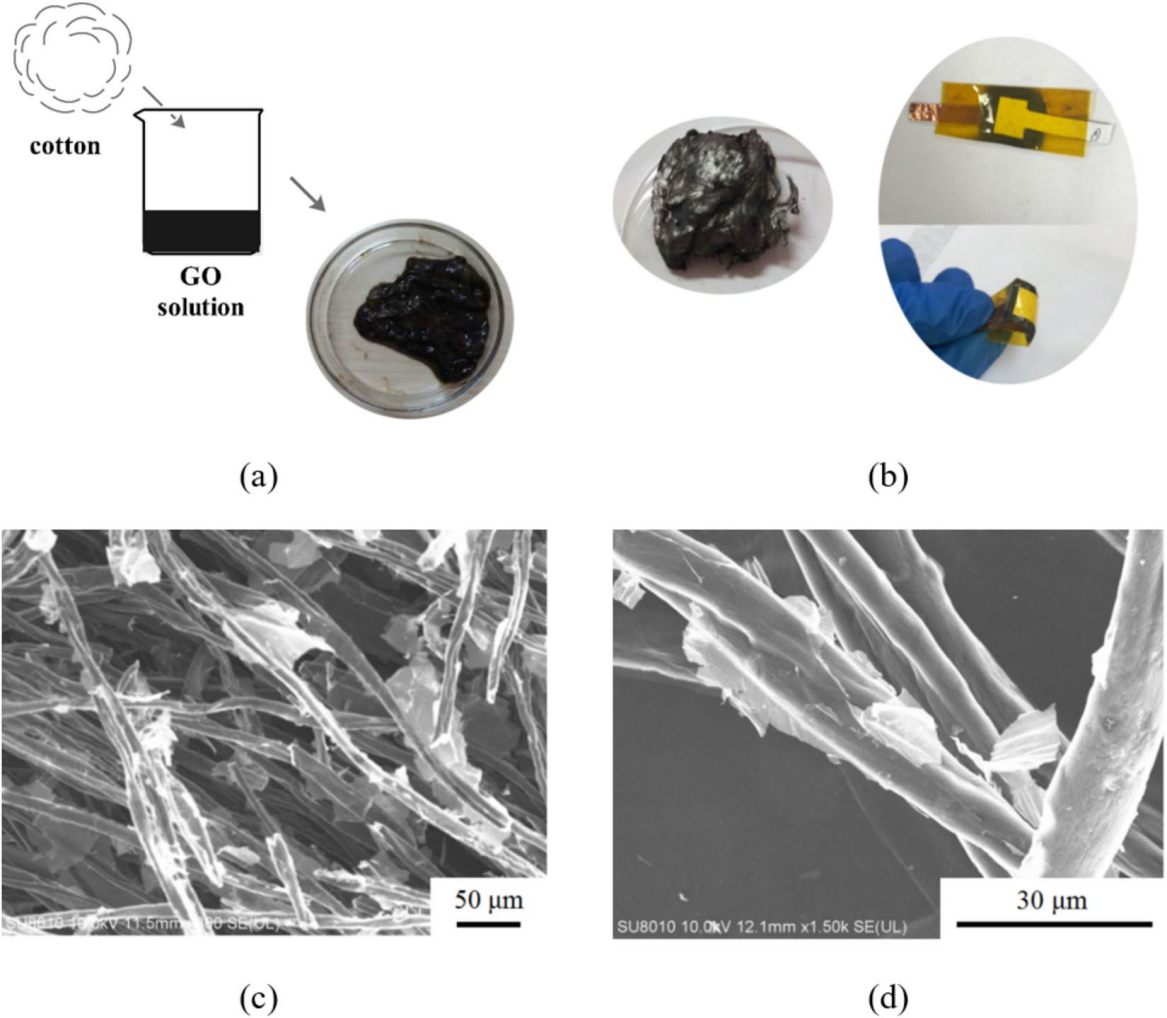
(**a**) Schematic of degreasing cotton pieces soaked in GO solution. (**b**) conductive rGO cotton and pressure sensors (**c**,**d**) SEM image showing how rGO was conformally adhered to cotton fibers.

Similar to graphene membranes, the rGO nanosheets are ultrathin and mechanically flexible, which allows them to conformally adhere to the surfaces of the cotton fibers. As shown in Fig. 1a,b, to obtain conductive cotton material, 2 mg/mL GO solution with dispersed GO sheets in aqueous solution was prepared. Subsequently, the degreasing cotton usually applied in laboratory was cut into small pieces and immersed in the GO solution for 12 h. The cotton pieces immersed in GO solution were then annealed in furnace at 250 °C for 5 h, under nitrogen protection. Thus the conductive cotton material was obtained. After being connected to copper electrodes and packaged with polyimide tape, the pressure sensor was constructed. Figure 1c,d show scanning electron microscopy (SEM) results of the rGO wrapped cotton, from which it can be clearly seen that the morphology of 3D porous networks and the cotton fibers were wrapped by the rGO sheets.

**Figure 2:**
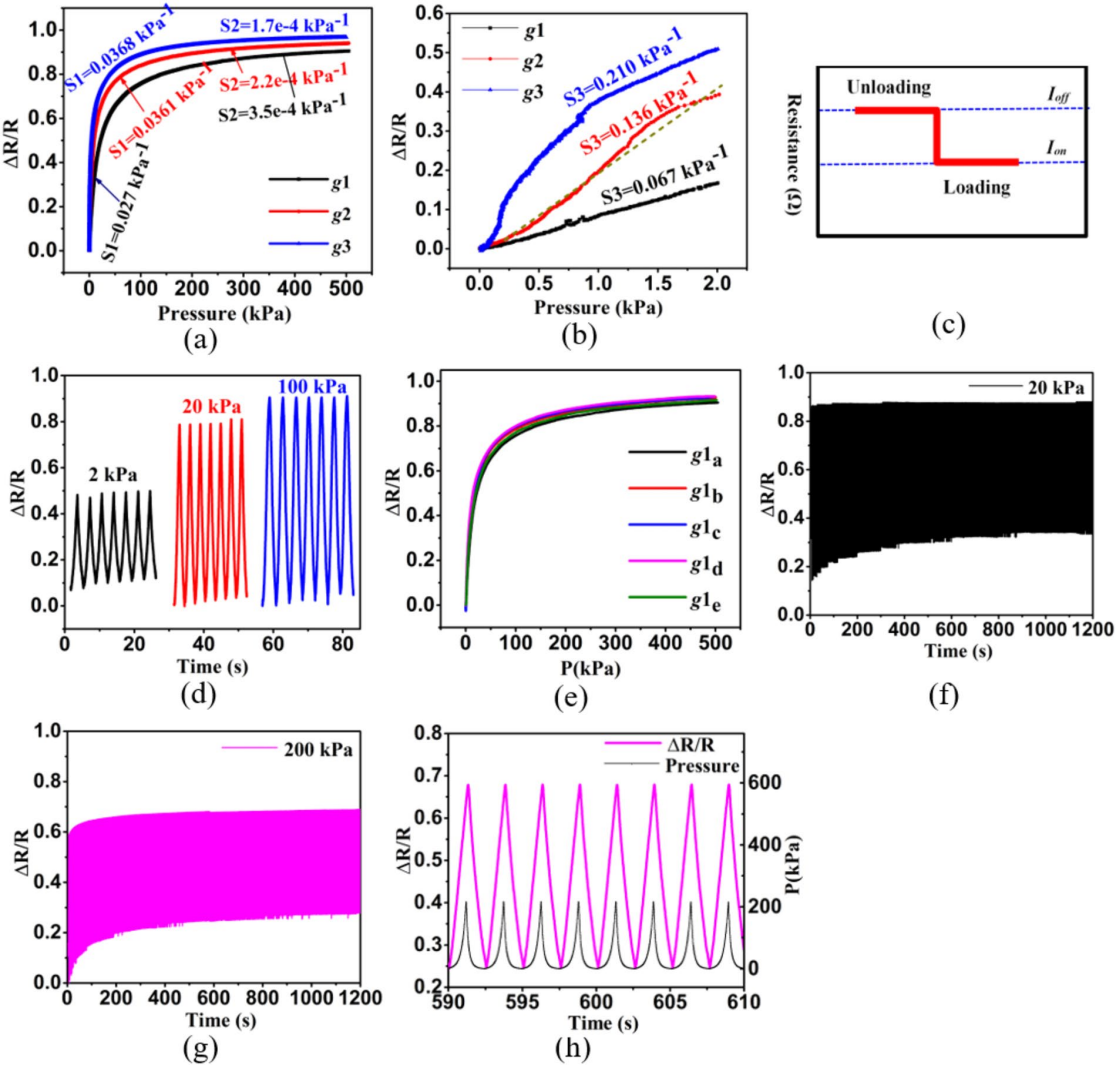
(**a**) Relative resistance change vs. pressure from 0 to 500 kPa of different sensors (*g*1, *g*2 and *g*3). (**b**) Relative resistance change vs. pressure from 0 to 2 kPa. (**c**) Sensing mechanism of how resistance changes with pressure in loading and unloading. (**d**) Relative resistance change of pressure of sensor *g*3 under different pressures. (**e**) Relative resistance change vs. pressure from 0 to 500 kPa of five pressure sensors *g*1_a_-*g*1_e_ with the same weight of 0.08 g. (**f**) Repeatability response of *g*2 in 500 loading-unloading cycles under the pressure of 20 kPa. (**g**) Repeatability response of *g*2 in 500 loading-unloading cycles under 200 kPa. (**h**) Enlarged view of (**g**) after about 250 cycles.

The full range (0–500 kPa) response properties of the conductive cotton based pressure sensor is shown in Fig. 2a, and the low pressure (0–2 kPa) response properties are shown in Fig. 2b. A testing platform was constructed, including a Shimadzu AGS-X mechanical testing machine and a Keithley 34410A digital multimeter, to study the performance of the pressure sensor developed in the present study. The sensitivity is defined as:

1 $$S=\delta (\Delta R/{R}_{0})/\delta P$$

2 $$\Delta R={R}_{0}-R$$

where *ΔR* represents the relative change of resistance value under pressure, *R* represents the resistance value when the pressure is implemented, *R*_0_ represents initial resistance value, and *δP* represents the variation of the applied pressure. As shown in Fig. 2a, the sensitivities of three different cotton based pressure sensors (*g*1, *g*2 and *g*3) were calculated with *S*_1_ indicating middle pressure range (0–20 kPa) and *S*_2_ indicating the high pressure range (100–500 kPa). Additionally, the sensitivities *S*_3_ are high in the very low range (0–2 kPa) of the sensors, and the linearity is also excellent, as shown in Fig. 2b. Figure 2c schematically demonstrates the working mechanism of the rGO modified cotton based pressure sensor. The SEM images in Fig. 1c,d above show the morphology of the 3D porous networks of the rGO wrapping cotton, suggesting there are a number of air gaps and overlapping in the networks structure of cotton. The rGO modified cotton based pressure sensor acts as a resistive pressure sensor. When the pressure is applied, air gaps reduce and overlapping in the cotton fibers increase, then the contact sites between the rGO nanosheets increase, and the resistance value of the sensor decreases.

Because there is no fixed shape in cotton fiber, the values of weight are used to differentiate the three samples. The weight of conductive cotton in *g*1, *g*2 and *g*3 sensors was measured by an electronic balance (LT1002E, with the accuracy of 0.01 g) and results were 0.08 g, 0.12 g and 0.14 g, respectively. The pressure sensors have a wide detection range and high sensitivity, and the sensitivities decline with the increase of the pressures. The performance of the conductive cotton pressure sensors varies with weight. As shown in Fig. 2a,b, when the weight of the conductive cotton increases, the sensitivity of the constructed sensors increases in the middle range and decreases in the high range. The 3D networks structure of cotton plays a key role in the excellent performance of the conductive cotton pressure sensor. This unique structure offers 3D conducting path, more air gaps and more contact sites than layer-structured materials like paper, which endows the graphene cotton based pressure sensor with a high sensitivity. Meanwhile, the 3D construction and high levels of flexibility of cotton fibers allow sensing material to withstand large loading, which endows the cotton based pressure sensor with an extremely wide detection range. The porous structure and air gaps lead to poor contact among the conducting material (rGO nanosheets) when there is no pressure, indicating that the pressure sensor has a high initial resistance. When a small pressure is applied, air gaps become smaller and the resistance of the graphene cotton based pressure sensor decreases sharply. There are more air gaps for the graphene cotton with greater weight and thickness. Therefore, the resistance of the sensor *g*3 decreases more quickly in the low pressure range, and *g*3 is more sensitive in the low pressure range of 0–20 kPa (0.21 kPa^−1^ in 0–2 kPa and 0.0368 kPa^−1^ in 2–20 kPa). Otherwise, when the applied pressure continuously increases, air gaps will disappear and the resistance of the graphene cotton based pressure sensor will decrease slowly. The sensor *g*1 is more sensitive in the high pressure range of 100–500 kPa and its value is 3.5 × 10^−4^ kPa^−1^.

The stability and repeatability are significant indicators for the performance of pressure sensors. Hence, a test was performed to detect the responses of the conductive cotton based pressure sensor under different loadings. As shown in Fig. 2d, the responses of pressure sensors to different pressures are stable and noise-free. The resistance of the sensor under middle pressure from 2 kPa to 20kPa changes more significantly than that under high pressure from 20 kPa to 100 kPa. Further, the long-time stability of the graphene cotton based pressure sensor was also tested. Figure 2e shows how the relative resistance of five different sensor samples *g*1_a_-*g*1_e_ with the same weight of 0.08 g changes with pressure from 0 to 500 kPa. The response performance of each sample is diverse, but the error is small and the sensitivities of the pressure sensors with their weight controlled only fluctuate within a certain range. Figure 2f,g show the repeatability responses of the pressure sensor *g*2 under pressure 20 kPa and 200 kPa in 500 loading-unloading cycles, it can be seen that the variation of relative resistance is almost constant throughout the cycling, except a slight increase in the initial resistance value. Figure 2h shows a local magnification of the repeatability response of *g*2 in 500 repetitive cycles under pressure of 200 kPa, the level of consistency of the response curve is good. These results confirm that the pressure sensors proposed in this study can maintain stability under repetitive loadings.

**Figure 3:**
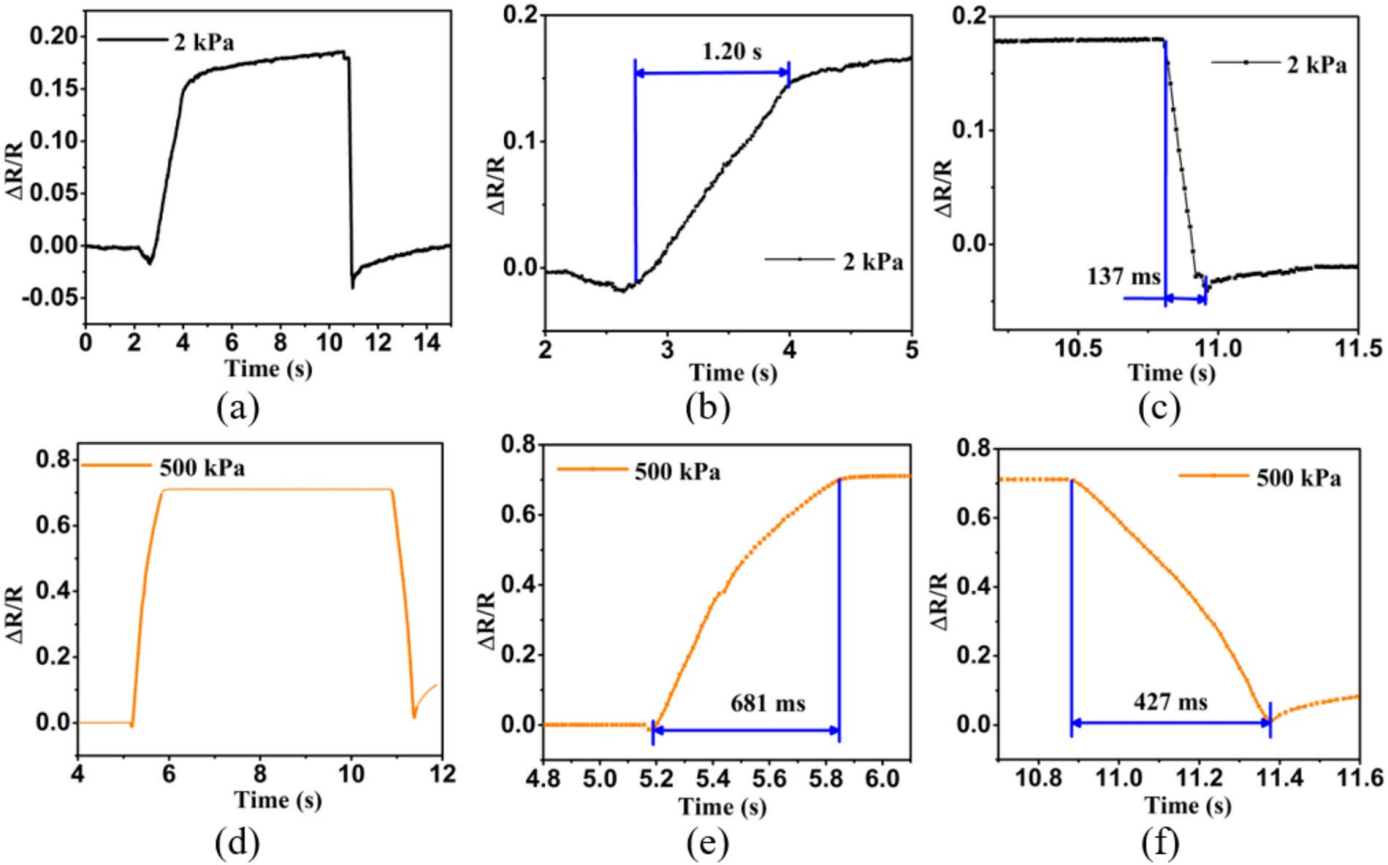
(**a**) Curve of a single cycle for *g*3 in loading-unloading test under 2 kPa. (**b**,**c**) Response time and recovery time of *g*3 under 2 kPa. (**d**) Curve of a single cycle of *g*3 in loading-unloading test under 500 kPa. (**e**,**f**) Response and recovery time of pressure sensor *g*3 under 500 kPa.

Furthermore, the response-recover properties of the pressure sensor were tested. Figure 3a shows a single response and recovery curve of sensor *g*3 with good reproducibility under the pressure of 2 kPa. According to Fig. 3,c, the response time and recovery time of the cotton based pressure sensor are 1200 ms and 137 ms, respectively. It turns out that the response-recovery time can change drastically under higher pressure. Figure 3d shows a single response and recovery curve of sensor *g*3 under the pressure of 500 kPa. As shown in Fig. 3e,f, the response time and recovery time of the pressure sensor are 681 ms and 427 ms, respectively. In addition, the response time is almost halved under high pressure, which may be attributed to the fact that larger deformation occurred in the conductive cotton when higher pressure was applied. And the recovery time increases to a certain extent under higher pressure as well, which leads to more recovery time for larger deformation. Also, as the cotton fiber has no fixed shape and errors exist in manual operation of different batches of annealing and packaging processes, the properties of the pressure sensors vary in different sensor samples, as shown in Fig. 2e. These results indicate good static and dynamic performance of the graphene cotton based pressure sensors proposed in this study, which well lends itself to human motion monitoring.

**Figure 4:**
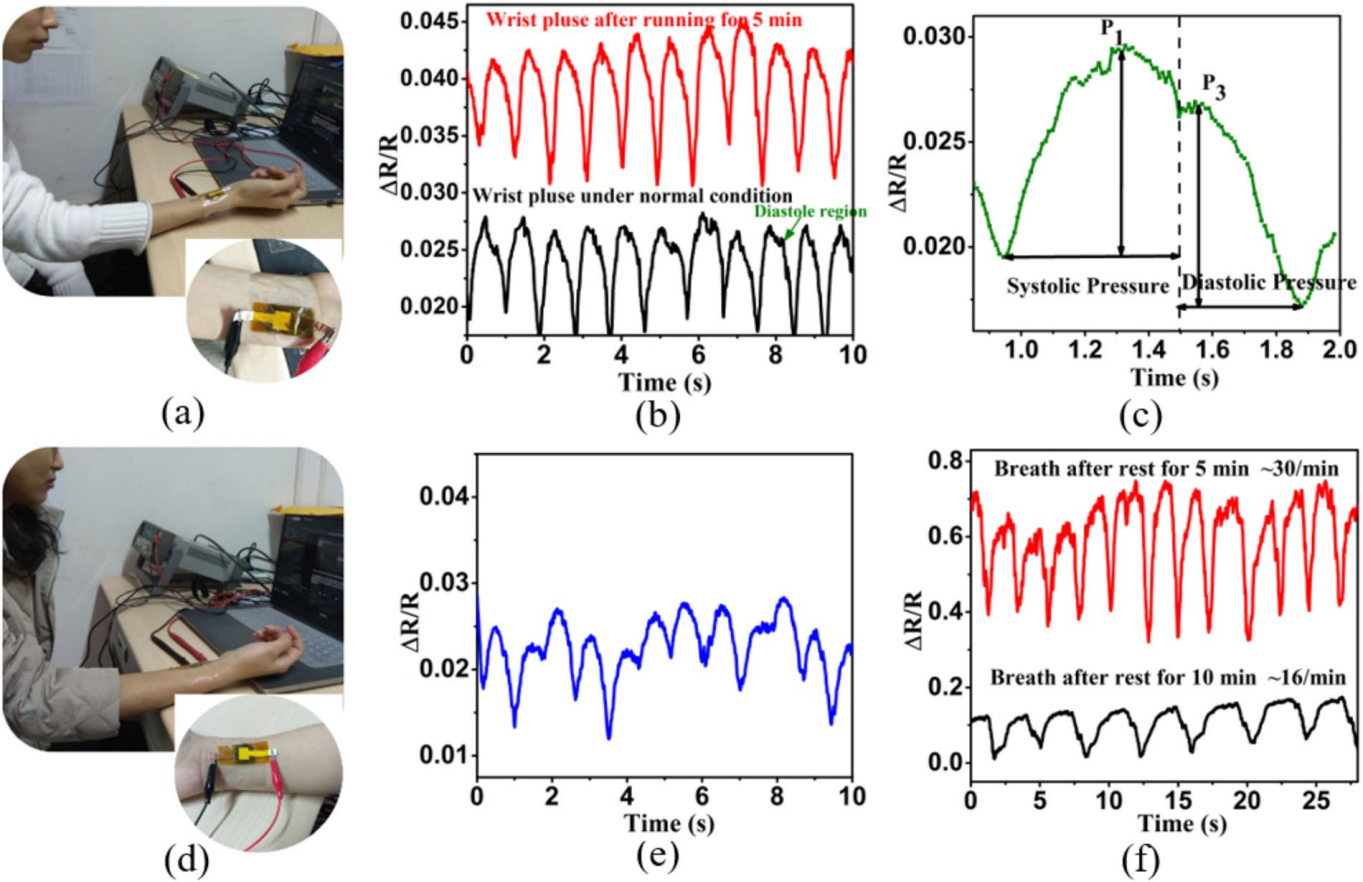
(**a**) Subject A taking the wrist pulse test. (**b**) Test result of subject A’s wrist pulse under normal condition and after he ran for 5 min (~1.2 km). (**c**) Enlarged view of diastole region in (**b**). (**d**) Subject B taking the wrist pulse test. (**e**) Test result of subject B’s wrist pulse under normal condition. (**f**) Subject A’s breath rate when he rested for 5 min and 10 min after running.

The real-time detection of human physiological signals is growingly prevalent in medical clinical diagnosis and human-computer interfaces. The conductive cotton based pressure sensor proposed in this study is attached to human wrists by tapes to realize the real-time monitoring of human pulse, as shown in Fig. 4a,d. Our testers included a boy (subject A, 25 years old, 185 cm height, 63 kg weight) and a girl (subject B, 23 years old, 158 cm height, 47.5 kg weight). According to the results shown in Fig. 4b,e, the pulse rates of subjects A and B are 64 bpm (beats per minute) and 70 bpm, respectively. Pulse beats of the boy are initially strong and then slightly slower, and pulse of the girl is initially weak and then slightly faster. The diastole region^31^ can be clearly seen in Fig. 4b,c, as marked in the pictures. For further comparison, the electrocardiography (ECG) examination was implemented for subjects A and B, the results show that the heart rates of subjects A and B are 65 bpm and 67 bpm, respectively (shown in Fig. S3a–c). As is commonly known, the heart rate and pulse rate are the same in a normal person. The results of our wrist pulse test by the cotton based pressure sensor are almost identical to those of ECG, which demonstrates the reliability of our wrist pulse test. Furthermore, the wrist pulse and respiratory rate of subject A were detected after he had ran for 5 min (for a distance of about 1.2 km), as shown in Fig. 4b,f. Because the boy’ body is very healthy, his heart rate recovered very fast after the run, so almost no change happened to his pulse rate, but his pulse amplitude increased significantly compared to the amplitude before running. Compared to heart rate, breath recovered more slowly, the boy’s respiration rate was ~30 times/min when he rested in sitting positions 5 min after running. And until 10 min after running, the boy’s breath returned to normal level (~16 times/min). The phenomenon observed in the experimental results are consistent with clinical medical knowledge. These experiments show the excellent performance of graphene cotton based pressure sensor in real-time monitoring of the physiological status of human bodies.

**Figure 5:**
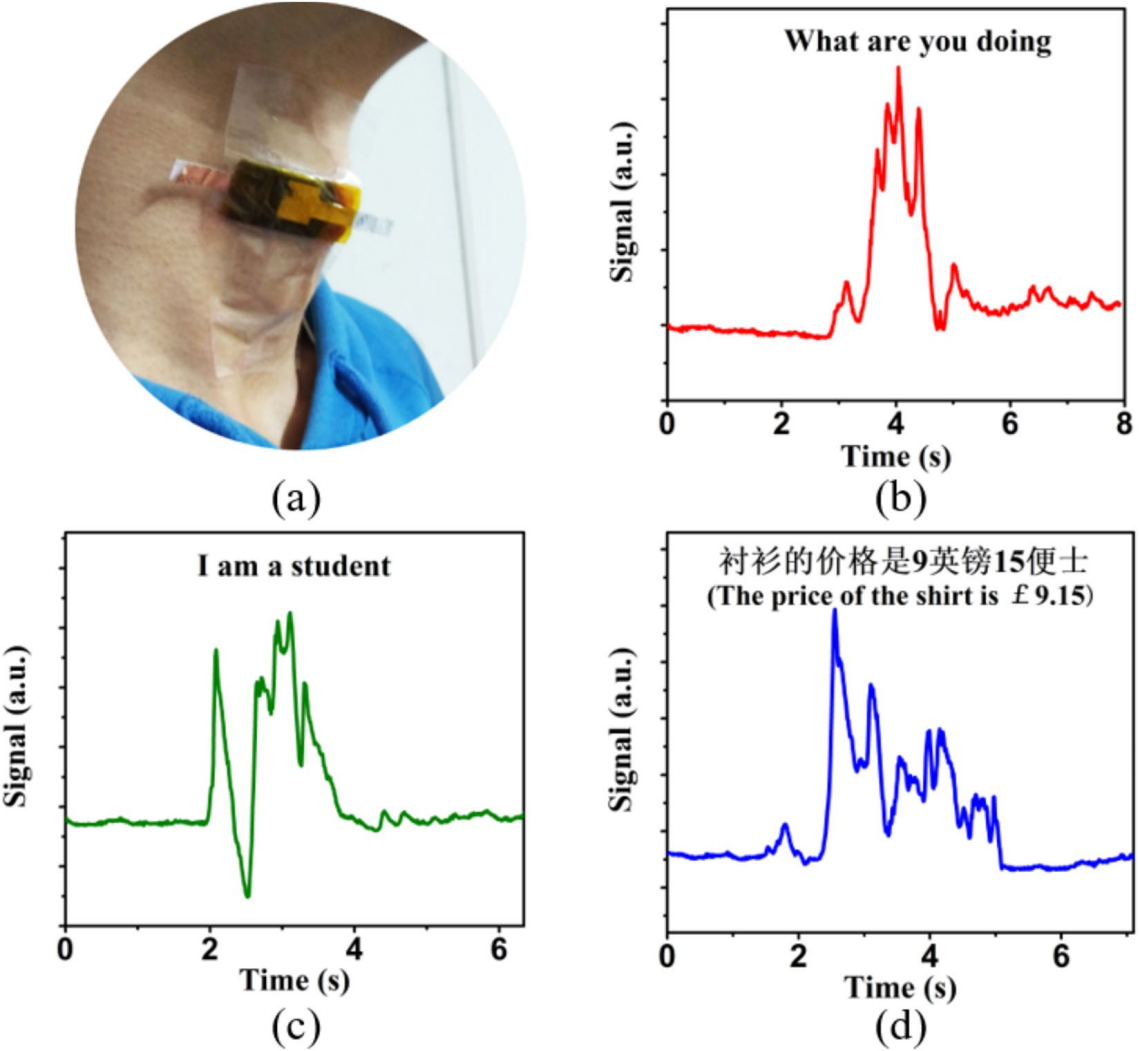
(**a**) The conductive cotton based pressure sensor applied in speech recognition. (**b**–**d**) Response curves of the tester when he spoke “I am a student”, “What are you doing” and “The price of the shirt is £ 9.15.” in Chinese.

The pressure sensor proposed in this study is also promising in speech recognition. The pressure sensor was attached to the throat of subject A by tapes, as shown in Fig. 5a. The muscle movements of the tester were detected while he spoke. Figure 5b–d show how the curves of resistance changed with time when the tester articulated different sentences, like “I am a student”, “What are you doing” and some Chinese words. Results indicate that the waveforms corresponding to different sentences are significantly different, and the difference in the pronunciation of each word is obvious and can be easily captured in the curves. And the reproducibility of the curves is good (shown in Fig. S4a,b). The results demonstrate that with high sensitivity and good reliability, the conductive cotton based pressure sensor can be well applied in speech recognition and other fields like human-computer interaction.

## Conclusion

In this study, the graphene modified conductive cotton was fabricated and applied in pressure detection. The rGO modified cotton based pressure sensor has a high sensitivity of 0.21 kPa^−1^ in a wide pressure range of up to 500 kPa. It also boasts good repeatability and stability. It demonstrates excellent performance in the detection of pulse, breath rate and muscle movement. The outstanding performance of the pressure sensor is attributed to the 3D porous networks structure of the cotton fibers. To sum up, the rGO modified cotton based pressure sensor proposed in this study have great promise in application of wearable devices especially in smart clothing.

## Methods

### Fabrication of rGO-cotton based pressure sensor

Absorbent cotton usually used in labs was cut into small pieces, and then immersed in 2 mg/mL graphene oxide (GO) aqueous solution for 12 h to make sure it was fully soaked by the solution. Subsequently, the cotton was put into chemical vapor deposition (CVD) furnace under 250 °C for 5 h, to reduce the GO dopant into rGO under nitrogen protection. The rGO cotton was not taken out until it was cooled down to room temperature within the furnace. Then the rGO cotton was sandwiched between the copper tapes as electrodes, and fixed by silver paste. The dimension of the top electrode was 1 cm × 1 cm. Finally, the cotton-based pressure sensor was packaged by polyimide tapes.

### Monitoring of human physiologic signals

The cotton-based pressure sensor was stuck to the wrists of subject A and B to detect their pulse, and stuck to the throat of subject A to detect his muscle movements while he was speaking. Resistance of the pressure sensor was measured by a Keithley 34410A digital multimeter throughout all the experiments. Informed consent to participation in the study and publication of identified information/images in an online open-access publication was obtained from all subjects prior to their enrollment in the study. This research was approved by the Ethics Committee of Xi’an Jiaotong University. All the experiments that were conducted on human wrists and necks in this study were performed in accordance with guidelines and regulations. No other human subjects were involved in our experiments or manuscript.

## Supplementary information

A wearable and sensitive graphene-cotton based pressure sensor for human physiological signals monitoring
