# Supplementary material for: A wearable and sensitive graphene-cotton based pressure sensor for human physiological signals monitoring
Source: Sci Rep. 2019 Oct 8;9:14457. doi: 10.1038/s41598-019-50997-1 (PMC6783539; doi:10.1038/s41598-019-50997-1)
Supplement: Supplementary file 1 — A wearable and sensitive graphene-cotton based pressure sensor for human physiological signals monitoring [file 41598_2019_50997_MOESM1_ESM.docx]

**A wearable and sensitive graphene-cotton based pressure sensor for human physiological signals monitoring**

**Ping LI,^a, b^ Libo ZHAO,^a, b*^ Zhuangde JIANG,^a,b^ Mingzhi Yu,^a,b^ Zhen LI,^a,b^ Xiangyang Zhou ^c*^ and Yulong ZHAO^a,b^**

^a.^State Key Laboratory for Manufacturing Systems Engineering, International Joint Laboratory for Micro/Nano Manufacturing and Measurement Technologies, Collaborative Innovation Center of Suzhou Nano Science and Technology, Xi'an Jiaotong University, Xi’an 710049, China. ^b.^School of Mechanical Engineering, Xi'an Jiaotong University, Xi’an 710049, China.

^c.^School of Instrumentation Science and Opto-electronics Engineering, Beihang University, Beijing 100191, China.

E-mail: [libozhao@mail.xjtu.edu.cn](mailto:libozhao@mail.xjtu.edu.cn) and xyzhou@buaa.edu.cn

**Table 1 Comparison of the proposed rGO-cotton based pressure sensor with other works before.**

| **Types of transduction** | **Materials** | **Sensitivity (kPa^-1^)** | **Pressure Range (kPa)** | **Ref.** |
| --- | --- | --- | --- | --- |
| piezoresistive | rGO-cotton | **0.21** | **0-2** | **This work** |
|  |  | **0.0368** | **2-20** |  |
|  |  | **0.0004** | **100-500** |  |
| triboelectric/ piezoelectric | PDMS/PDMS-carbon nanotube | 0.5 V/kPa | 5–450 | [^1^](#_ENREF_1) |
| piezoresistive | graphene porous network - PDMS | 0.09 | 0-1000 | [^2^](#_ENREF_2) |
|  |  | / | 1000-2000 |  |
| piezoresistive | hollow-sphere polypyrrole | 133.1 | < 30 Pa | [^3^](#_ENREF_3) |
|  |  | / | 0-5 |  |
| piezoresistive | gold nanowire- tissue paper | 1.14 | 0-5 | [^4^](#_ENREF_4) |
|  |  | / | 5-50 |  |
| capacitive | PDMS-coated conductive fiber | 0.21 | 0-2 | [^5^](#_ENREF_5) |
|  |  | 0.064 | 2-10 |  |
| piezoresistive | reduced graphene oxide foam | 15.2 | 0-300 Pa | [^6^](#_ENREF_6) |
|  |  | / | 300 Pa-10 kPa |  |
| piezoresistive | rGO–PU | 0.26 | 0–2 | [^7^](#_ENREF_7) |
|  |  | 0.03 | 2-10 |  |
| piezoresistive | laser-scribed graphene (LSG) | 0.96 | 0-50 | [^8^](#_ENREF_8) |
|  |  | 0.005 | 50-113 |  |
| piezoresistive | rGO-P(VDF-TrFe) | 3.1 | 0-20 | [^9^](#_ENREF_9) |
|  |  | 15.6 | 20-55 |  |
| tunneling/heterostructure | graphene/BN | relatively low | ~5 GPa | [^10^](#_ENREF_10) |

**Fig. S1 The Raman spectrum of the rGO wrapped on cotton fibers.**

| 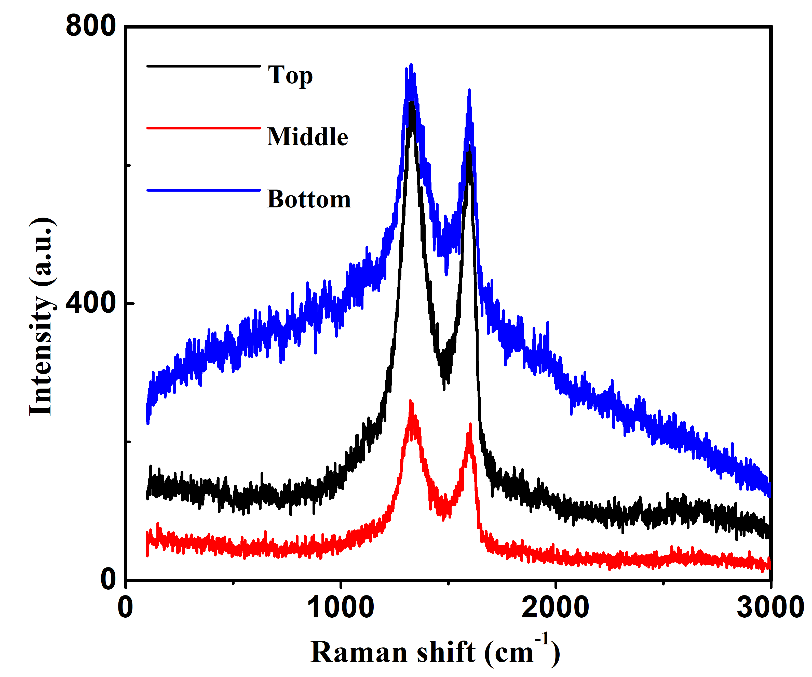 |
| --- |

**PS:** The density of the rGO sheets was highest on the top surface of the cotton fibers, so the peak of Raman shift was most obvious (the black curve); The bottom surface of the cotton took second place (the blue curve); And the rGO density in the middle layer of the cotton fibers was smallest, so the peak of Raman shift was very low (the red curve).

**Fig. S2 The resistance value of the rGO-cotton based pressure sensors changed with the pressure loading.**

| 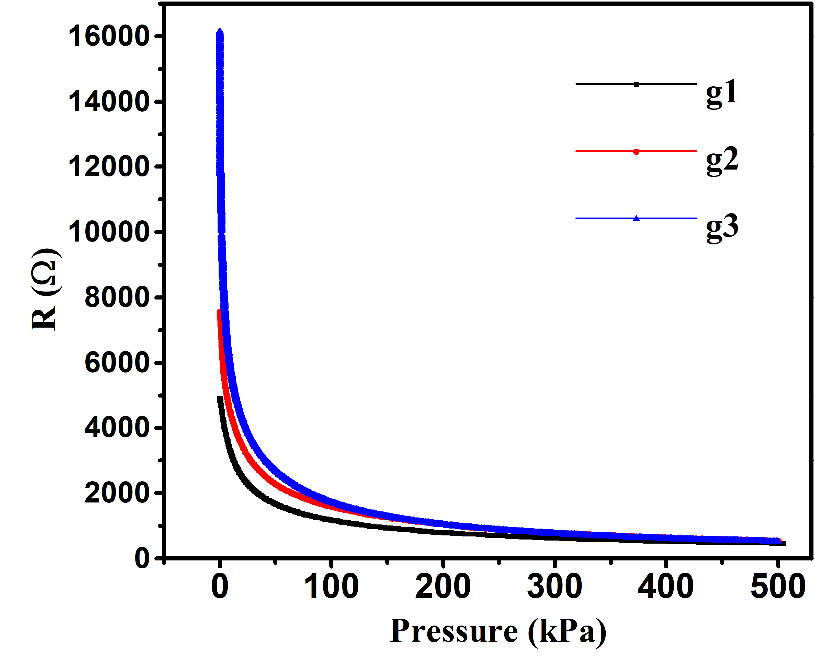 |
| --- |

**Fig. S3 The** **electrocardiography (ECG) examination for** **subjects A and B. (a)** **Picture of ECD testing of subject A. (b)** **ECG test result of subject A, the heart rate is 65 bpm. (c) ECG test result of subject B, the heart rate is 67 bpm.
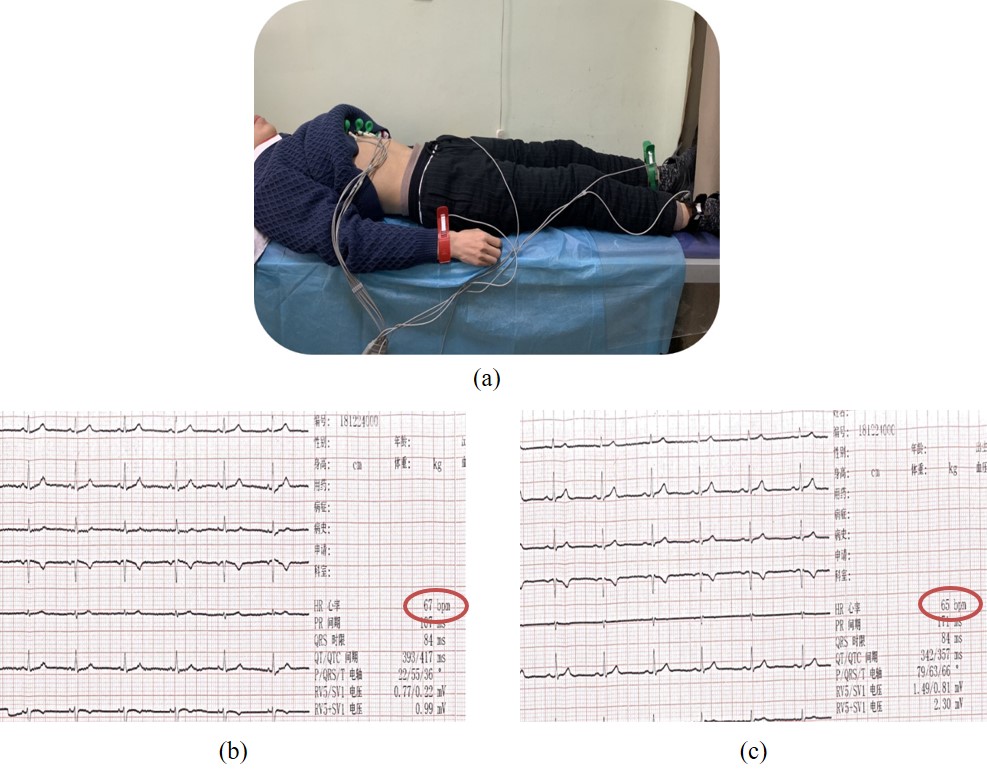
**

**Fig. S4 The reproducibility tests for rGO-cotton based pressure sensor applied in speech recognition: (a) Response curves when subject A speaking “What are you doing” repeatedly. (b) Response curves for “I am a student” repeatedly.**

**
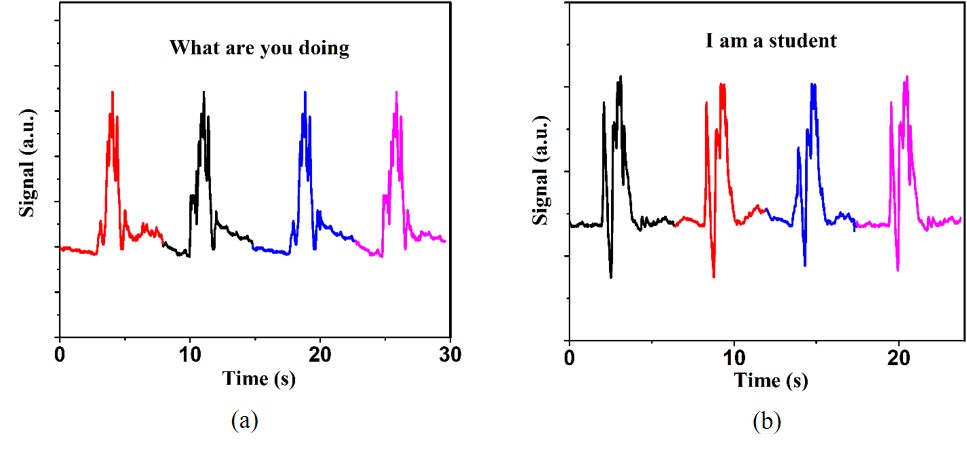
**

**REFERENCE**

1 Rasel, M. S. *et al.* An impedance tunable and highly efficient triboelectric nanogenerator for large-scale, ultra-sensitive pressure sensing applications. *Nano Energy* (2018).

2 Pang, Y. *et al.* A Flexible, Highly Sensitive and Wearable Pressure and Strain Sensors with Graphene Porous Network Structure. *Acs Applied Materials & Interfaces* **8** (2016).

3 Pan, L. *et al.* An ultra-sensitive resistive pressure sensor based on hollow-sphere microstructure induced elasticity in conducting polymer film. *Nature Communications* **5**, 3002 (2014).

4 Gong, S. *et al.* A wearable and highly sensitive pressure sensor with ultrathin gold nanowires. *Nature Communications* **5**, 3132 (2014).

5 Lee, J. *et al.* Conductive fiber-based ultrasensitive textile pressure sensor for wearable electronics. *Advanced Materials* **27**, 2433-2439 (2015).

6 Hou, C., Wang, H., Zhang, Q., Li, Y. & Zhu, M. Highly conductive, flexible, and compressible all-graphene passive electronic skin for sensing human touch. *Advanced Materials* **26**, 5018-5024 (2014).

7 Yao, H. B. *et al.* A flexible and highly pressure-sensitive graphene-polyurethane sponge based on fractured microstructure design. *Advanced Materials* **25**, 6692-6698 (2013).

8 Tian, H. *et al.* A Graphene-Based Resistive Pressure Sensor with Record-High Sensitivity in a Wide Pressure Range. *Sci Rep* **5**, 8603 (2015).

9 Lou, Z., Chen, S., Wang, L., Jiang, K. & Shen, G. An ultra-sensitive and rapid response speed graphene pressure sensors for electronic skin and health monitoring. *Nano Energy* **23**, 7-14 (2016).

10 Xu, Y. *et al.* In-plane and tunneling pressure sensors based on graphene/hexagonal boron nitride heterostructures. *Applied Physics Letters* **99**, 197 (2011).
